# The Relevance of Food Constituents to the Irritable Bowel Syndrome: A Rome IV-Based Prevalence Study Among Medical Students

**DOI:** 10.5152/tjg.2023.22490

**Published:** 2023-08-01

**Authors:** Ahmed H. Mujamammi, Rasha Al-Hamdan, Essa M. Sabi, Zyad A. Aldosari, Abdullah M. Shadid, Abdulrahman Shadid, Salman Alagla, Hameed S. Humaid, Talal Abozaid, Nahla Azzam

**Affiliations:** 1 Division of Clinical Biochemistry, Department of Pathology, King Saud University Faculty of Medicine, Riyadh, Saudi Arabia; 2 Department of Community Health Sciences, King Saudi University Faculty of Applied Medical Sciences, Riyadh, Saudi Arabia; 3 Department of MedicineKing Saudi University Faculty of Medicine, Riyadh, Saudi Arabia; 4 Division of Gastroenterology, Department of Medicine, King Saud University Faculty of Medicine, Riyadh, Saudi Arabia

**Keywords:** Food, dietary intake, irritable bowel syndrome, prevalence

## Abstract

**Background/Aims:**

Irritable bowel syndrome is prevalent in the general population. This study investigates the association between dietary intake and irritable bowel syndrome in medical college students at King Saud University besides its prevalence.

**Materials and Methods:**

This is an analytical cross-sectional study of 426 students (271 males and 155 females, age 21.21 ± 1.58 years) from 5 academic levels of King Saud University Medical College. A self-reported questionnaire for Rome IV criteria was completed by each participant. They also filled out a food frequency questionnaire to assess their nutritional intake.

**Results:**

The overall prevalence of irritable bowel syndrome was 17.8% without correlation to age and academic year in Medical School. However, the prevalence was higher in females than in males (40/115 vs. 36/235, *P *= .001). The irritable bowel syndrome group consumed significantly more energy, carbohydrates, and saturated fatty acids, while the non-irritable bowel syndrome group consumed significantly more fibers and niacin (*P* < .001 and *P* = .005, respectively).

**Conclusion:**

About 17.8% of medical students had irritable bowel syndrome with a greater prevalence in females. The irritable bowel syndrome group consumed significantly more energy, carbohydrates, and saturated fatty acids, while the non-irritable bowel syndrome group consumed significantly more fibers and niacin. Our results did not show any significant association between irritable bowel syndrome and fermentable oligosaccharide, disaccharide, monosaccharide, and polyol intake. Overall, both groups were not adhering to the Saudi dietary recommended intake.

**Main Points**

- Irritable bowel syndrome (IBS) is common among medical students. In our survey, 17.8% of medical students described symptoms of IBS according to Rome IV criteria, with greater prevalence among females.
- In our study, the IBS group reported to significantly consume more energy, carbohydrates, and Saturated fatty acids. Moreover, this group significantly consumed less fibers and niacin when compared to the non-IBS group.
- The findings shed light on the association between IBS and dietary intake among Saudi medical students. A large cohort study is recommended to give a better understanding of the relationship of IBS and Saudi diet.

## Introduction

Irritable bowel syndrome (IBS) is one of the common disorders of gut–brain interactions (previously called “functional gastrointestinal disorders”), with a global prevalence of 9.2%, which varies between 0.2% and 29.2% according to country^1^ and ranges between 9.3% and 43.5% among medical institutions around the globe.^2^ The method of diagnosis utilized has an impact on this IBS prevalence.

Irritable bowel syndrome is characterized by chronic or recurrent stomach pain that is relieved or made worse by defection or by a change in bowel habits,^3,4^ and its pathophysiology is complex and incompletely understood.^5^ The most common method for making a differential diagnosis based on these symptoms is the Rome IV criteria.^6^

Irritable bowel syndrome negatively impacts the quality of life and work productivity, as it has been estimated that patients would give up 10-15 years of life expectancy for an immediate cure^7^ or would accept a hypothetical medication’s 99% chance of curing their symptoms in exchange for a median 1% risk of sudden death.^8^ In addition, there has been an overall increase in the length of physician visits associated with IBS,^9^ and the estimated direct care costs for IBS range from £45.6 to £200 million annually in the UKto €3.1 to €4.1 billion in Germany.^7^

Generally, genetics,^10,11^ gender,^12,13^ anxiety, depression,^14,15^ smoking,^16^ and diet^17,18^ have been regarded as IBS risk factors. Particularly, the pathophysiology of IBS has been closely linked to diet or nutritional consumption.^5^ Despite the numerous IBS studies that have been conducted since the publication of the Rome IV criteria in 2016,^6^ there are limited studies that look at the prevalence of IBS in both the Saudi general population and medical students using the Rome IV criteria. Furthermore, there is also less research on the association between IBS and food.^2,19,20^

We investigated the association between dietary intake and IBS among medical college students at King Saud University, Saudi Arabia. The prevalence of IBS was also reported.

## Materials and Methods

### Subjects

In this analytical cross-sectional study, a total of 426 students (2-grade levels of preclinical years and 3-grade levels of clinical years) at King Saud University, Medical College in Riyadh, Saudi Arabia, were studied from November 2020 to December 2020. A stratified sampling method was used to calculate the sample size. Stratification considered the gender and academic year; this stratification was done to eliminate any confounder related to gender and academic year. Medical students were randomly chosen from the students’ list provided by King Saud University Medical Students Council and were invited to complete an online self-reported questionnaire via email due to coronavirus-19 precautions, and all survey answers were collected anonymously without identification information. However, the excluded participants in this study involved the students who refused to give (online) informed consent for participation and those who self‐reported having the following criteria: diabetes mellitus, celiac disease, inflammatory bowel disease (Crohn and Ulcerative Colitis), cancer anywhere in the gastrointestinal tract, and current infection of the gastrointestinal tract. We obtained informed consent from all participants. Each analysis and questionnaire was completed in accordance with the rules and approval of the Institutional Review Board of King Saud University Medical City, Riyadh, Saudi Arabia (KSU-IRB 017E).

The sample size calculation was based on a recently published study on medical students, their sample size was 232, and the prevalence of IBS was 31%,^16^ so a proportion of 31% was taken with an alpha (*α*) of 0.05 and a precision of 5%. Thus, the estimated sample size was 329 participants. An additional 20% was added to compensate for any nonresponses or excluded participants. This resulted in a total sample size of 395.

### Protocol

The first part included informed consent and demographic details such as age, gender, academic year, and smoking habits.

The second part contains an English version of the Rome IV criteria questionnaire.^21^ It also includes the presence and frequency of abdominal pain or discomfort, its onset, the connection between the pain and the frequency or type of stools, and whether the symptoms got better after defecation. The diagnosis of IBS required that students reported not only abdominal pain or discomfort at least 1 day a week during the past 3 months but also at least 2 of the following 3 symptoms: pain is related to defecation, onset is associated with a change in stool frequency, and a change in stool appearance. Criteria must be fulfilled for the last 3 months with symptom onset at least 6 months before diagnosis. The questionnaire also classifies subjects into the following subgroups: constipation-predominant IBS (IBS-C), diarrhea-predominant IBS (IBS-D), mixed IBS (IBS-M), or the subject never or rarely has abnormal stools (IBS-U), based on patient report of the usual consistency of abnormal stools by using a picture of the Bristol Stool Scale.

The third part included a food frequency questionnaire (FFQ) to validate the dietary intake in the Arabic language.^22^ The survey measures the consumption of food during the past year. The amount of food is what is consumed on average. Consisted of 140 food items developed to obtain the dietary habits of Saudis. This FFQ had been tested for its validity, internal consistency, test–retest reliability, and completeness of the food list. After that, the nutrient content of each item was calculated. These values included the amount of not only energy, proteins, fat, and carbohydrates but also of fermentable oligosaccharides, disaccharides, monosaccharides, and polyols (FODMAPs) (i.e., fructose, lactose, polyols, sorbitol, mannitol, fructans, galactans, raffinose, and stachyose), calcium, phosphate, iron, zinc, vitamin A, B1, B2, B,6, C, E, niacin, and folate. Dietary intake was calculated using the USDA software (18th-2st ED, 2009, 2010) program.

## Statistical Analysis

We compared the gender, grade level, and food constituents between non-IBS and IBS groups, using the chi-square test and independent samples *t*-tests. Data were expressed as a frequency or mean with a SD. Two-tailed *P* <.05 were considered to indicate statistical significance. Statistical analyses were performed using Statistical Package of Social Sciences (SPSS) version 25 (IBM Corp.; Armonk, NY, USA).

## Results

### Student Characteristics and Prevalence of Irritable Bowel Syndrome

Of 445 responses collected, 19 were later excluded for meeting one of the exclusion criteria. The final analysis included 426 responses. The mean age of the included students was 21.21 ± 1.58 (range 18-27), and 271 (63.6%) were males. Seventy-six students (36 male and 40 female) had symptoms of IBS. Therefore, the overall prevalence of IBS was 17.8% without correlation to age and academic year in Medical School. The female group showed a higher prevalence (40/155, 26%) than the male group (36/271, 13.3%). Prevalence of IBS during the 2 preclinical and 3 clinical years was 10.8%, 18.4%, 16.5%, 18.4%, and 25%, respectively, and showed no difference among each academic level. The IBS group was further subdivided into diarrhea-predominant (IBS-D), constipation-predominant (IBS-C), along with diarrhea and constipation (IBS-M), or undetermined categories (IBS-U). Among the 76 IBS students, the proportions of IBS-D, IBS-C, IBS-M, and IBS-U were 4.5%, 3.1%, 9.9%, and 0.5%, respectively (Table 1).

Female participants were significantly more affected by IBS than male participants (*P* = .001, odds ratio = 2.2705; 95% CI = 1.374, 3.753); hence being a female is a risk factor for IBS. Analysis of the smoking status (*P* = .052, OR = 2.154; 95% CI = 0.979, 4.737) and the academic year (*P* = .147, OR = 0.675; 95% CI = 0.395, 1.15) showed no significant differences (Table 2).

### Irritable Bowel Syndrome and Nutritional Intake

In the current study, the energy intake showed a significant difference between IBS and non-IBS groups with a mean of 2.158 kilocal and 1.820 kilocal, respectively (*P* = .001, 95% CI = 0.1402, 0.5356). The IBS group also consumed significantly higher carbohydrates (*P* = .029, 95% CI = .230, 0.4224) and saturated fatty acids (SFA) (*P* = .003, 95% CI = .1105, .5274). Furthermore, the IBS group consumed significantly less fibers than non-IBS group with a mean of 1.553 g and 1.909 g, respectively (*P* < .001, 95% CI = 0.1402, 0.5356) and niacin with a mean of 1.158 mg and 1.337 mg, respectively (*P* = .005, 95% CI = −0.30, −0.06).

Compared to individuals without IBS, those with IBS had similar intakes of all the other analyzed micro- and macronutrients. They consumed similar proteins, fats, and unsaturated fatty acids (*P* = .074, *P* = .079, and *P* = .297, respectively). The consumption of the minerals calcium, potassium, phosphorus, iron, iodine, and zinc was also not significantly different between the 2 groups (*P* = .533, *P* = .836, *P* = .973, *P* = .965, *P* = .776, and *P* = .351, respectively). They also had no significant difference in intake of vitamin A, vitamin B1, vitamin B2, vitamin B6, vitamin C, vitamin E, and folate (*P* = .873, *P* = .356, *P* = .627, *P* = .152, *P* = .623, *P* = .395, and *P* = .114, respectively). With regard to FODMAPs, there were no significant differences in the consumption of fructose, lactose, polyols, sorbitol, mannitol, fructans, galactans, raffinose, and stachyose (*P* = .266, *P* = .167, *P* = .586, *P* = .718, *P* = .979, *P* = .806, *P* = .875, *P* = .875, and *P *= .381, respectively) (Table 3).

Overall, on average, only 22.56% from the IBS group and 27.25% from the non-IBS group were following the Saudi recommended dietary allowance.

## Discussion

In the current study, the overall prevalence rate of IBS among medical students was 17.8%, which is higher than the pooled global prevalence, which was 9.2%,^1^ and the overall prevalence of Saudi undergraduate students. However, it is lower than what has been reported by local studies among medical students.^16,23,24^

Many studies have observed that the prevalence of IBS is higher in females.^1^ It has been suggested that sex hormones and pain perception play a potential role in these gender differences.^12,25,26^ In our study, the prevalence of IBS was significantly higher among females, which is consistent with the literature.

The prevalence of IBS appeared to be influenced by the academic year at college. However, a strong connection between the 2 has not yet been well established. Our study showed no significant difference between preclinical and clinical students, which is consistent with several international studies.^27,28^ On the other hand, a study conducted among medical students and interns from King Abdulaziz University Hospital, Jeddah, showed the prevalence is significantly higher among higher academic years and suggested that the reason is an increased study and work stressors.^29^ In contrast, a systemic review conducted in Iran concluded that IBS is more prevalent among the first and second years.^30^

Despite the adverse effects of smoking,^31^ our results showed that smoking status had no significant association with IBS prevalence. This could be due to the small number of smokers in our study for both IBS and non-IBS groups, which were 10 (13.16%) and 23 (6.6%), respectively. This result concedes with a study conducted in Jeddah^29^ and another study conducted in Malaysia,^32^ both among medical students. Nonetheless, a recent study among Saudi undergraduate students concluded that cigarette smoking is a risk factor for IBS.^15^

It is well established that diet plays an essential role in the pathophysiology of IBS.^6,33,34^ Many studies associated worsening IBS symptoms with particular kinds of diet, such as high in fat and carbohydrates,^35-37^ spicy food,^38^ and caffeine.^39^ However, other studies associated improvement of symptoms and quality of life with a diet low in FODMAPs.^40-44^ In our region, a study conducted among nurses at King Abdulaziz University Hospital to determine the prevalence, severity, and predictors of IBS concluded that the first predictor of IBS was food hypersensitivity.^18^ Moreover, another study found that specific diets, especially garlic, onions, and coffee, were found to increase the IBS symptoms. On the other hand, decreasing carbohydrates and increasing fiber would enhance the patient’s health and decrease the symptoms.^19^

In the current study, medical students showing symptoms of IBS demonstrated significant differences in energy intake compared to the non-IBS group, which is consistent with a recent North American population-based study.^45^ Also, intake of carbohydrates, SFA, fiber, and niacin showed significant differences. Carbohydrates and SFA is linked to alterations in microbiota, inflamed microenvironment, and overgrowth of harmful bacterial species.^46,47^

These effects on gut flora are important in the pathophysiology of IBS.^48^ Though both groups consumed more carbohydrates and SFA than the recommended dietary allowance (DRA), our results showed that the IBS group consumed them significantly more compared to the non-IBS group, which may be associated with their symptoms. On the other hand, dietary fiber appears to improve the global symptoms of IBS,^49^ as it acts as a prebiotic to intestinal microbiota that induces the growth of beneficial bacteria;^50^ however, our findings show that both study groups consumed dietary fiber below the DRA with the consumption among IBS group being significantly less. In regard to niacin, there is growing evidence that highlights the importance of niacin in neuronal health^51^ and the digestive system.^52^ The relationship between IBS and niacin should be further studied. Contrary to expectations, this research did not find any significant difference between FODMAPs intake and IBS. 

This study has several limitations, which should be considered in future research. First, individuals with IBS were identified through Rome IV criteria and not through proper evaluation by a physician. Another potential limitation is recall bias. Finally, the study is done in only 1 of the 21 medical colleges in Saudi Arabia.

## Conclusion

In conclusion, 17.8% of medical students have IBS, with a greater prevalence in females. The IBS group consumed significantly more energy, carbohydrates, and SFA, while the non-IBS group consumed significantly more fibers and niacin. Our results did not show any significant association between IBS and FODMAP intake. Overall, both groups were not adhering to the Saudi DRA.

More research utilizing the Rome IV criteria is required to assess the prevalence of IBS in the general Saudi population and in specific groups. Since Saudi food differs greatly from Western cultures, a large cohort study regarding the dietary or nutritional pattern and etiology of IBS is essential.

## Figures and Tables

**Table 1.** Socio-Demographic and Clinical Characteristics of Patients with Irritable Bowel Syndrome (n = 426)

Variable	Item Mean (SD)
Age	21.21 (1.58)
	n (%)
Gender (male)	271 (63.6)
Academic year:	
First year	84 (19.7)
Second year	76 (17.8)
Third year	91 (21.4)
Fourth year	87 (20.4)
Fifth year	88 (20.7)
Smoking status (yes)	33 (7.7)
IBS status (yes)	76 (17.8)
IBS subtype:	
IBS-C	13 (3.1)
IBS-D	19 (4.5)
IBS-M	42 (9.9)
IBS-U	2 (0.5)

IBS, irritable bowel syndrome; IBS-C, constipation predominant; IBS-D, diarrhea predominant; IBS-M, mixed diarrhea and constipation; IBS-U, undetermined categories.

**Table 2.** Association of Categorical Study Variables with Irritable Bowel Syndrome Status

Variables	IBS Status (%)		Chi-square value	*P*^b^	OR	95% CI of OR
	IBS (n = 76, 17.8%)^a^	Non-IBS (n = 350, 82.2%)				
Gender						
Male	36 (13.3%)	235 (86.7%)	10.549	0.001	2.2705	1.374-3.753
Female	40 (25.8%)	115 (74.2%)				
Smoking						
Yes	10 (13.2%)	23 (6.6%)	3.79	0.052	2.154	0.979--4.737
No	66 (86.8%)	327 (93.4%)				
Academic year						
Basic years	23 (30.3%)	137 (39.1%)	2.099	0.147	0.675	0.395- 1.15
Clinical years	53 (69.7%)	213 (60.9%)				

^a^Prevalence of IBS; ^b^analyzed by Pearson chi-square test.

Basic years: first and second years, clinical years: third, fourth, and fifth years; IBS, irritable bowel syndrome.

**Table 3.** Comparison of 2 Groups (Irritable Bowel Syndrome and Non-Irritable Bowel Syndrome) with Respect to the Level of Nutritional Intake

Study Variable	IBS Group (n = 76) Mean (±SD)	Non-IBS Group (n = 350) Mean (±SD)	Difference in Means (±SD)	*t*-value	*P*	95% CI
Protein (g)	2.224 (±0.8262)	2.034 (±0.8389)	0.1894 (±0.1059)	1.789	.074	(−0.02, 0.3975)
Carbohydrate (g)	2.368 (±0.7974)	2.146 (±0.8039)	0.2227 (±0.1016)	2.192	.029	(0.230, 0.4224)
Fats (g)	2.237 (±0.8774)	2.040 (±0.8852)	0.1968 (±0.1118)	1.760	.079	(−0.02, 0.4167)
Energy (kcal)	2.158 (±0.8335)	1.820 (±0.7861)	0.3379 (±0.1006)	3.360	.001	(0.1402, 0.5356)
SFA (g)	2.579 (±0.7351)	2.260 (±0.8587)	0.3189 (±0.1061)	3.007	.003	(0.1105, 0.5274)
USFA (g)	1.618 (±0.7826)	1.717 (±0.7398)	-0.10 (±0.0946)	−1.0	.297	(−0.28, 0.0872)
Fiber (g)	1.553 (±0.7553)	1.909 (±0.7814)	-0.36 (±0.0983)	−3.6	<.001	(−0.55, −0.16)
Calcium (mg)	1.724 (±0.8884)	1.794 (±0.8944)	-0.07 (±0.1131)	−0.062	.533	(−0.29, 0.1516)
Potassium (mg)	1.447 (±0.7553)	1.429 (±0.7096)	0.0188 (±0.0908)	0.207	.836	(−0.16, 0.1974)
Phosphorus (mg)	1.434 (±0.6992)	1.437 (±0.6773)	0.00 (±0.0862)	−0.03	.973	(−0.17, 0.1667)
Iron (mg)	1.487 (±0.7210)	1.483 (±0.7130)	0.0040 (±0.0904)	0.044	.965	(−0.17, 0.1817)
Iodine (ug)	1.342 (±0.6230)	1.320 (±0.6107)	0.0221 (±0.0776)	0.285	.776	(−0.13, 0.1746)
Zinc (mg)	1.224 (±0.5316)	1.291 (±0.5821)	-0.07 (±0.0726)	−0.93	.351	(−0.21, 0.0749)
Vitamin A (ug)	1.618 (±0.7995)	1.634 (±0.7779)	-0.02 (±0.0989)	−0.16	.873	(−0.21, 0.1786)
Vitamin B1 (mg)	1.263 (±0.5971)	1.334 (±0.6101)	-0.07 (±0.0769)	−0.92	.356	(−0.22, 0.0801)
Vitamin B2 (mg)	1.211 (±0.5245)	1.243 (±0.5254)	-0.03 (±0.0665)	−0.49	.627	(−0.16, 0.0983)
Vitamin B6 (ug)	1.263 (±0.5743)	1.374 (±0.6196)	-0.11 (±0.0774)	−1.4	.152	(−0.26, 0.0411)
Vitamin C (mg)	1.421 (±0.6785)	1.463 (±0.6708)	-0.04 (±0.0851)	−0.49	.623	(−0.21, 0.1254)
Vitamin E (mg)	1.224 (±0.5561)	1.286 (±0.5801)	-0.06 (±0.0729)	−0.85	.395	(−0.21, 0.0812)
Niacin (mg)	1.158 (±0.4337)	1.337 (±0.5084)	-0.18 (±0.0628)	−2.9	.005	(−0.30, −0.06)
Folate (ug)	1.221 (±0.4984)	1.320 (±0.5567)	-0.11 (±0.0692)	−1.6	.114	(−0.25, 0.0226)
Fructose (g)	2.145 (±0.8749)	2.031 (±0.7879)	0.1133 (±0.1017)	1.114	.266	(−0.09, 0.3133)
Lactose (g)	2.145 (±0.8749)	2.003 (±0.7958)	0.1419 (±0.1025)	1.384	.167	(−0.06, 0.3434)
Polyols (g)	2.145 (±0.7951)	2.094 (±0.7179)	0.0505 (±0.0927)	0.544	.586	(−0.13, 0.2326)
Sorbitol (g)	1.789 (±0.8377)	1.751 (±0.8316)	0.0380 (±0. 1054)	0.361	.718	(−0.17, 0.2452)
Mannitol (g)	1.868 (±0.9429)	1.871 (±0.9135)	0.00 (±0.1163)	−0.03	.979	(−0.23, 0.2255)
Fructans (g)	2.105 (±0.8419)	2.080 (±0.8078)	0.0253 (±0.1030)	0.245	.806	(−0.18, 0.2277)
Galactans (g)	1.974 (±0.8637)	1. 957 (±0.8229)	0.0165 (±0.1051)	0.157	.875	(−0.19, 0.2231)
Raffinose (g)	2.053 (±0.8148)	2.037 (±0.7655)	0.0155 (±0.0980)	0.158	.875	(−0.18, 0.2081)
Stachyose (g)	2.105 (±0.8259)	2.020 (±0.7549)	0.0853 (±0.0972)	0.877	.381	(−0.11, 0.2763)

*t*-Test for 2 means has been employed with the following considerations: df = 424, *P* ≤.05 is considered statistically significant for a 2-tailed test.

energy, total intake of calories; IBS, irritable bowel syndrome; SFA, saturated fatty acids; USFA, unsaturated fatty acids.
